# Supplementary material for: Successful Incorporation of Exosome-Capturing Antibody-siRNA Complexes into Multiple Myeloma Cells and Suppression of Targeted mRNA Transcripts
Source: Cancers (Basel). 2022 Jan 23;14(3):566. doi: 10.3390/cancers14030566 (PMC8833399; doi:10.3390/cancers14030566)
Supplement: Supplementary file 1 [file cancers-14-00566-s001.zip › cancers-1516107-supplementary.pdf]

cancers MDPI

Supplementary Materials

Successful Incorporation of Exosome-capturing Antibody-siRNA Complexes into Multiple Myeloma Cells and Suppression of Targeted mRNA Transcripts

Emi Soma, Asako Yamayoshi, Yuki Toda, Yuji Mishima, Shigekuni Hosogi and Eishi Ashihara

Table of Contents

Figure S1. Confocal microscopic images of anti-CD63 mAb-branched Arg conjugated siRNA complexes transferred into NCI-H929 cells.
Figure S2. Evaluation of the efficacies of siRNAs against *MYC* and *CTNNB1*.
Figure S3. Western Blot.
Table S1. Sequences of siRNAs used in this study.
Table S2. Sequences of primers used for qRT-PCR in this study.

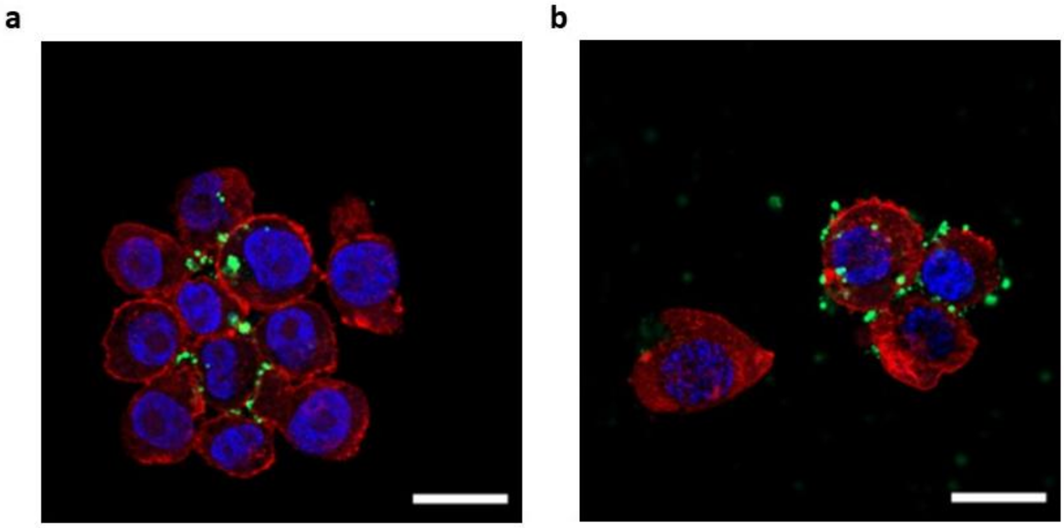

Figure S1. Confocal microscopic images of anti-CD63 mAb-branched Arg conjugated siRNA complexes transferred into NCI-H929 cells. NCI-H929 cells were treated with anti-CD63 mAb-conjugated siRNA labeled with fluorescence at anti-CD63 mAb-Arg linker- siRNA ratios of 1:1 (300:300 nM) (**a**) and 5:1 (1500:300 nM) (**b**). Scale bars, 20 μm. Red, rhodamine-phalloidin; blue, Hoechst 33342 (nuclei); green, FITC-labeled siRNAs.

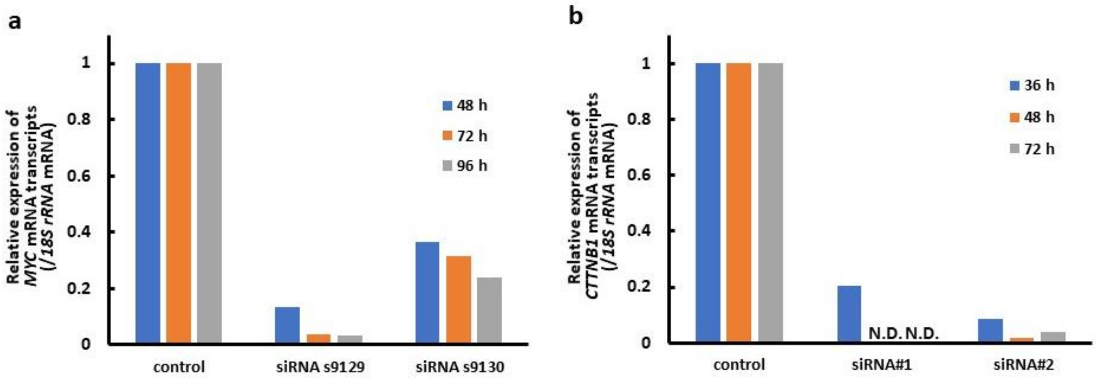

Figure S2. Evaluation of the efficacies of siRNAs against *MYC* and *CTNNB1*. (**a**) Both siRNAs targeting *MYC* effectively suppressed *MYC* mRNA transcripts in HeLa cells; siRNA s9129 was more effective than siRNA s9130. The optimal duration for silencing *MYC* mRNA transcripts by siRNA s9129 was 96 h. (**b**) Both siRNAs targeting *CTNNB1* effectively suppressed *CTNNB1* mRNA transcripts in HeLa cells; siRNA #2 was more effective than siRNA #2. The optimal duration for silencing *CTNNB1* mRNA transcripts by siRNA #1 was 48 h. N.D., not done.

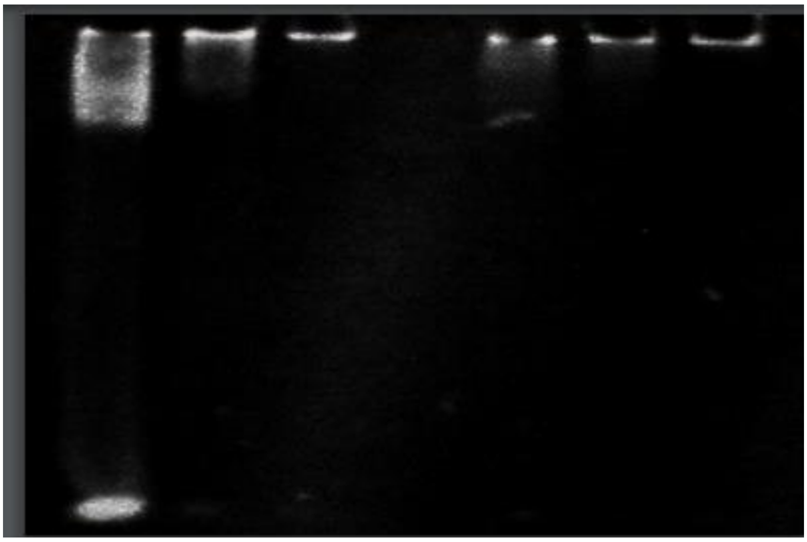

Figure S3. Western Blot.

Table S1. Sequences of siRNAs used in this study.

Target	Orientation	Length (bp)	Sequence
Luc2	Forward	21	5'-AUUGAAUCUUAUAGUCUUGCA-3'
	Reverse	21	5'-CAAGACUAUAAGAUUCAAUCU-3'
Luc+	Forward	21	5'-UCUUCAUAGCCUUAUGCAGUU-3'
	Reverse	21	5'-CUGCAUAAGGCUAUGAAGAGA-3'
F-*luc2*	Forward	21	5'-AUUGAAUCUUAUAGUCUUGCA-3'
	Reverse	20	5'-F- CAAGACUAUAAGAUUCAAUCU-3'
MYC s9129	Forward	20	5'-AGACCUUCAUCAAAAAACAU-3'
	Reverse	19	5'-AUGUUUUUGAUGAAGGUCU-3'
MYC s9130	Forward	19	5'-GAGCUAAAACGGAGCUUUU-3'
	Reverse	18	5'-AAAAGCUCCGUUUUAGCU-3'
CTNNB1 siRNA#1	Forward	21	5'-P-GCUUUAGUAAAUAUAAUGAGG-3'
	Reverse	21	5'-P-UCAUUAUAUUUACUAAAGCUU-3'
CTNNB1 siRNA #2	Forward	21	5'-P-GUUGUAAACUUGAUUAACUAU-3'
	Reverse	21	5'-P-AGUUAAUCAAGUUUACAACUG-3'

bp, base pair; _, 2'-OMe modification; _, locked nucleic acid (LNA) modification; _, LNA with methylcytosine modification; ▯, 2'-fluoro modification; P, 5'-phosphorylated modification.

Table S2. Sequences of primers used for qRT-PCR in this study.

Target	Orientation	Length (bp)	Sequence	Probe number
c-MYC	Forward	20	5'-GCTGCTTAGACGCTGGATTT-3'	#66
	Reverse	18	5'-TAACGTTGCGGGGCTTCG-3'	
CTNNB1	Forward	20	5'-GCTTTCAGTTGAGCTGACCA-3'	#21
	Reverse	20	5'-CAAGTCCAAGATCAGCAGTCTC-3'	
18S rRNA	Forward	20	5'-GCAATTATTCCCCATGAACG-3'	#48
	Reverse	20	5'-GGGACTTAATCAACGCAAGC-3'	

bp, base pair.
